# Supplementary material for: Microstructure arrays of DNA using topographic control
Source: Nat Commun. 2019 Jun 7;10:2512. doi: 10.1038/s41467-019-10540-2 (PMC6555807; doi:10.1038/s41467-019-10540-2)
Supplement: Supplementary file 1 — Supplementary Information [file 41467_2019_10540_MOESM1_ESM.pdf]

Supplementary Information

Microstructure arrays of DNA using topographic control

Yun Jeong Cha[1†], *Soon Mo Park*[1†], *Ra You*[1], *Hyoungsoo Kim*[2] and *Dong Ki Yoon*[1,3]*

[1] Graduate School of Nanoscience and Technology, Korea Advanced Institute of Science and Technology, Daejeon 34141, Republic of Korea

[2] Department of Mechanical Engineering, Korea Advanced Institute of Science and Technology (KAIST), Daejeon 34141, Republic of Korea

[3] Department of Chemistry and KINC, Korea Advanced Institute of Science and Technology, Daejeon 34141, Republic of Korea

†These authors contributed equally to this work.

*To whom correspondence should be addressed.
E-mail: nandk@kaist.ac.kr

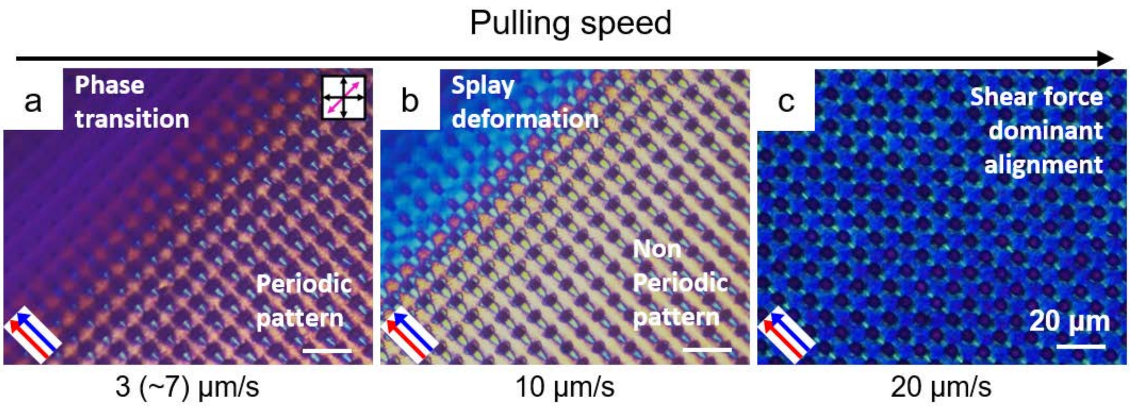

Supplementary Figure 1. DNA microstructure-generation depending on the pulling speed, (a) 3~7 μm/s. (b) 10 μm/s, and (c) 20 μm/s. All scales are 20 μm.

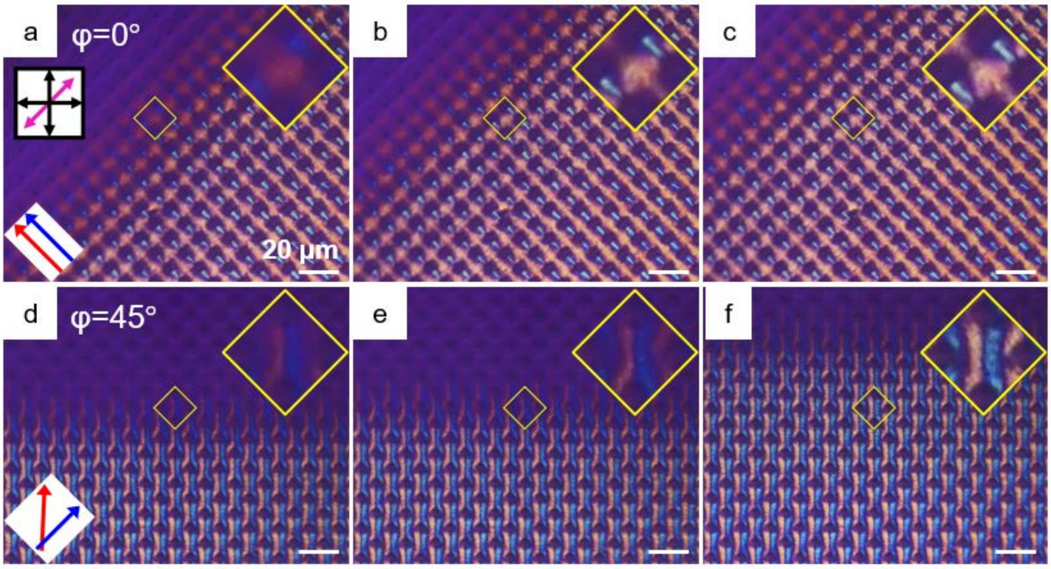

Supplementary Figure 2. Sequential growth of DNA microstructure at $\varphi = 0°$ (a-c), $\varphi = 45°$ (d-f) rotated ±45° based on the original position. All scales are 20 μm.

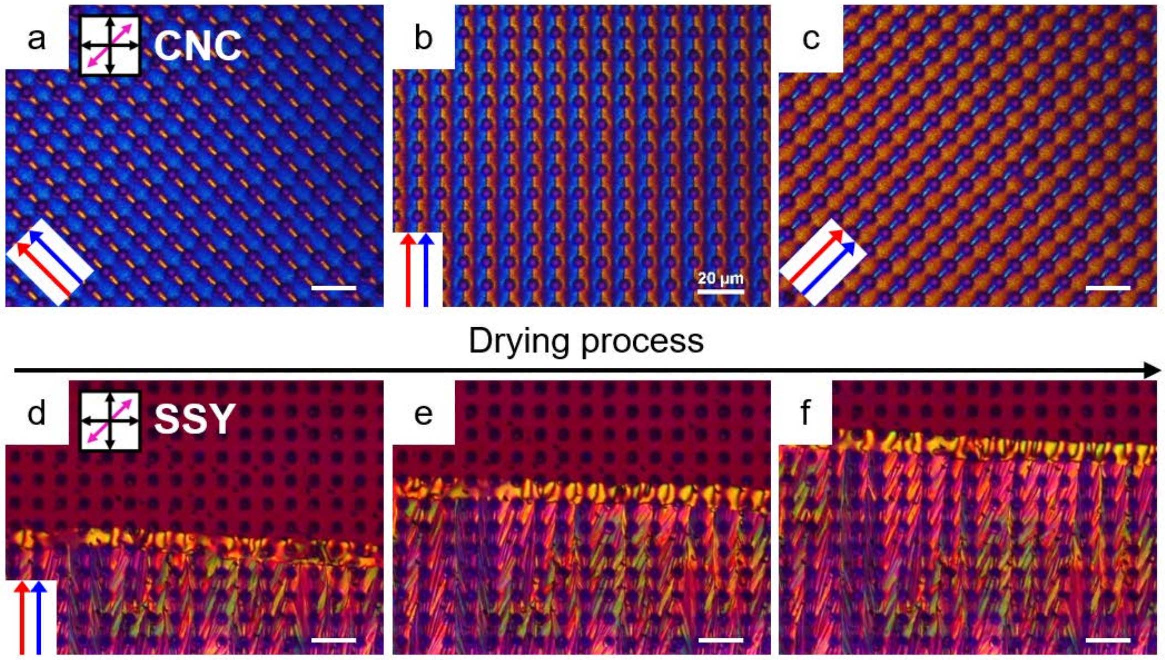

Supplementary Figure 3. Fabricated cellulose nanocrystal (CNC) microstructures using a concentration of 80 mg/ml at (b) $\varphi = 0°$ and by (a,c) rotating sample ±45°; (d-f) Drying process of sunset yellow (SSY) solution using a concentration of 350 mg/ml at $\varphi = 0°$ does not show the similar behavior with DNA's case. All scales are 20 µm.

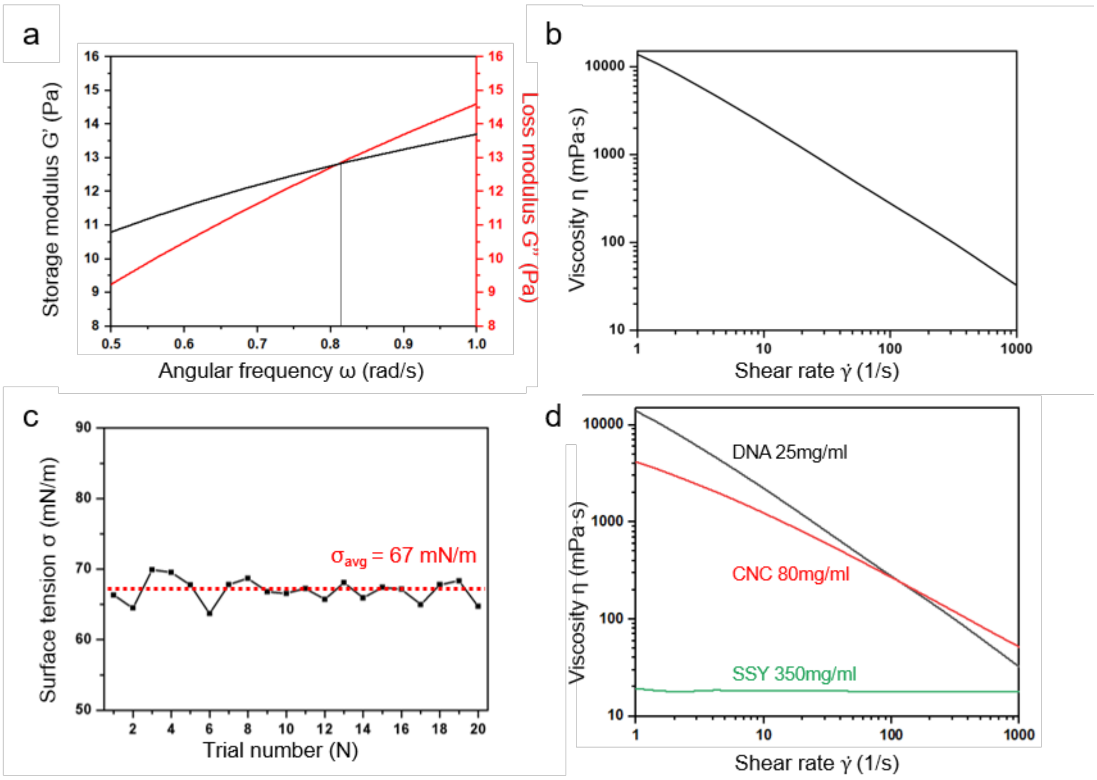

Supplementary Figure 4. (a) Rheometer measurement of storage (G') and loss modulus (G''), (b) shear rate-viscosity curve and (c) surface tension of 25 mg/ml DNA solution. (d) Comparison of shear-viscosity curves for DNA, CNC, and SSY. The results present that CNC and DNA show a shear-thinning behavior while SSY shows a Newtonian behaviour.

Material	Persistence length (λ_p)	Contour length (L)	Diameter (D)	Aspect ratio (L/D) & (λ_p/D)	Flexibility (λ_p/L)
Salmon sperm DNA (DNA)	50nm	680nm	2nm	340(25)	Semiflexible
Sunset Yellow FCF (SSY)	10nm	≥10nm	1nm	≥10(10)	Semiflexible
Cellulose nanocrystal (CNC)	150nm	300nm	10nm	30(15)	Semiflexible

Supplementary Table 1. Molecular scale properties of salmon sperm DNA (DNA), sunset yellow FCF (SSY), and cellulose nanocrystal (CNC).
