# Supplementary material for: Microstructure arrays of DNA using topographic control
Source: Nat Commun. 2019 Jun 7;10:2512. doi: 10.1038/s41467-019-10540-2 (PMC6555807; doi:10.1038/s41467-019-10540-2)
Supplement: Supplementary file 3 — Description of Additional Supplementary Files [file 41467_2019_10540_MOESM3_ESM.pdf]

Description of Additional Supplementary Files

File Name: Supplementary Movie 1
Description: In-situ growing sequences in Fig. 2

File Name: Supplementary Movie 2
Description: In-situ growing sequences in Fig. 3

File Name: Supplementary Movie 3
Description: In-situ growing sequences in Supplementary Fig. 2
